# The Prevalence of Prior Mental Health and Substance Use Problems in Older Persons and Their Families

**DOI:** 10.1093/geroni/igaa057.552

**Published:** 2020-12-16

**Authors:** Todd Becker, John Cagle, Paul Sacco

**Affiliations:** 1 University of Maryland, Baltimore, Baltimore, Maryland, United States; 2 University of Maryland, Baltimore, Maryland, United States

## Abstract

Although research has shown mental health and substance use problems (MHSUPs) are fairly prevalent in older adults (OAs), less research has considered MHSUPs in hospice beneficiaries and their families. This secondary analysis filled this gap using the Health and Retirement Study’s Core survey wave from 2014 and Exit wave data from 2016. These data are nationally-representative of OAs aged 50+. Each biennial wave introduces an experimental module to a random 10% of Core survey participants. One Core 2014 experimental module included self-report indicators assessing past MHSUPs, like depression and anxiety, using single items. Exit 2016 proxy-reported information about respondent deaths was used to create a decedent subsample. Descriptive statistics established MHSUP prevalence rates in OAs and their family. The self-report depression indicator was validated against the 8-item Center for Epidemiological Studies Depression Scale (CESD-8) at the ≥3, ≥4, and ≥5 cut points using χ2 analyses. The full sample’s (N=1,461) average age was 68 years. Participants were mostly non-Hispanic (87.5%), White (72.8%), and female (59.7%). The decedent subsample (n=64) was bifurcated by hospice (54.7%) versus nonhospice (45.3%) utilization. Most participants in the full sample (63.9%), hospice decedent subsample (77.1%), and nonhospice decedent subsample (75.9%) endorsed at least one MHSUP. Depression and anxiety were the most common MHSUPs in each study sample. The CESD-8 was associated with the self-report depression indicator across all cut points (p<.001). Practitioners, policymakers, and researchers should consider the high prevalence rates of MHSUPs found in OAs and their families when designing programs, policies, and research.

